# NT-proBNP, Cardiometabolic Risk Factors, and Nutritional Status in Hemodialysis Patients

**DOI:** 10.1155/2017/1312547

**Published:** 2017-09-17

**Authors:** Jacques Ducros, Laurent Larifla, Henri Merault, Lydia Foucan

**Affiliations:** ^1^Centre de Dialyse AUDRA, Hôpital RICOU, Pointe-à-Pitre, Guadeloupe, France; ^2^Service de Néphrologie, Centre Hospitalier Universitaire, Guadeloupe, France; ^3^Service de Cardiologie, Centre Hospitalier Universitaire, Guadeloupe, France; ^4^Equipe de Recherche Epidémiologie Clinique et Médecine sur le Risque Cardio Métabolique, ECM/LAMIA, EA 4540, Université des Antilles, Centre Hospitalier Universitaire, Guadeloupe, France

## Abstract

**Background:**

We aimed to evaluate the association between NT-proBNP and malnutrition in HD patients while taking into account the four established categories of parameters for diagnosis of protein energy wasting (PEW).

**Methods:**

A cross-sectional study was performed in Afro-Caribbean dialysis patients. One component in each of the 4 categories for the wasting syndrome was retained: serum albumin ≤ 38 g/L, BMI ≤ 23 Kg/m^2^, serum creatinine ≤ 818 *µ*mol/L, and normalized protein catabolic rate (nPCR) ≤ 0.8 g/kg/day. NT-proBNP was assessed using a chemiluminescence immunoassay. Two multivariate logistic regression models were performed to determine the parameters associated with high NT-proBNP concentrations.

**Results:**

In 207 HD patients, 16.9% had PEW (at least three components). LVEF lower than 60% was found in 13.8% of patients. NT-proBNP levels ranged from 125 to 33144 pg/mL. In model 1, high levels of NT-proBNP (≥6243 pg/mL) were independently associated with PEW OR 14.2 (3.25–62.4), male gender 2.80 (1.22–6.57), hsCRP > 5 mg/L 3.90 (1.77–8.57), and dialysis vintage > 3 years 3.84 (1.35–10.8). In model 2, LVEF OR was 0.93 (0.88–0.98). NT-proBNP concentrations were significantly higher when the PEW component number was higher.

**Conclusion:**

In dialysis patients, high NT-proBNP levels must draw attention to cardiac function but also to nutritional status.

## 1. Introduction

Uremic malnutrition, also called, protein energy wasting (PEW), corresponding to a decrease in energy and body protein, is a common problem in patients with end stage renal disease (ESRD) undergoing hemodialysis (HD) [1–3]. This syndrome found approximately in 20 to 70% of HD patients [1] has been associated with inflammation [4], overhydration [5], and high morbidity and mortality [2, 6]. Previous studies have also reported association between N-terminal pro-brain natriuretic peptide (NT-proBNP) levels and malnutrition assessed using the subjective global assessment and malnutrition-inflammation score [7, 8] and it was suggested that PEW might have a direct effect on the level of NT-proBNP by affecting ventricular remodeling in HD patients [7]. The International Society of Renal Nutrition and Metabolism (ISRNM) proposed, in 2008, a uniformed nomenclature to define malnutrition in individuals with kidney disease [3] from several parameters among four established categories (biochemical criteria; body mass and composition, muscle mass, and dietary intakes). The severity of malnutrition can then be identified according to the number of malnutrition parameters.

Brain natriuretic peptide (BNP), one member of the natriuretic family, is synthesized by ventricular cardiomyocytes, in response to wall stress, and plays a major role in regulation of blood pressure and extracellular volume [9]. In the circulation, the enzyme-mediated cleavage of proBNP results in BNP, the active peptide, and NT-proBNP, an inactive N-terminal fragment. NT-proBNP is cleared essentially by the kidney, while BNP is cleared by its specific natriuretic peptide receptors and by an endopeptidase, independently of glomerular filtration rate [10–12]. Blood concentration of NT-proBNP has been associated with left ventricular disorders, hypervolemia [13, 14], and identified as a predictive factor of cardiac events and mortality in the HD population [13–15].

Since NT-proBNP level is directly influenced by kidney function, elevated levels of this inactive fragment are often observed in HD patients without clinical evidence of cardiovascular disease [15, 16].

The question of whether NT-proBNP is a marker of malnutrition in HD is still asked. Thus, we tested the hypothesis that NT-proBNP concentrations vary according to the number of malnutrition markers.

## 2. Objectives

We aimed to evaluate the association between NT-proBNP and malnutrition taking into account the four categories of the ISRMN definition for PEW and to analyze the relationships between NT-proBNP concentrations and the number of malnutrition markers in HD patients.

## 3. Subjects and Methods

This study was approved by the Institutional Review Board Committee of the dialysis centre which has waived the need for informed consent since the current study reported the results of the annual checkup of HD patients.

### 3.1. Patient Population

In a cross-sectional study, we included Afro-Caribbean patients who underwent maintenance HD treatment for more than three months and who were checked in December 2015 in the AUDRA centre (one of the dialysis facilities in the island of Guadeloupe, France). For the purpose of the study, patients included had no acute cardiac insufficiency, acute coronary complication, or chronic obstructive pulmonary disease.

Standard dialysis treatment consisted of three weekly sessions using bicarbonate buffer and synthetic high flux membrane. The dialysate electrolytes prescription usually includes sodium (140 mmol/L), potassium (2 mmol/L), bicarbonates (35 mmol/L), and calcium (1.5 mmol/L). Sodium prescription is adapted according to blood pressure and fluid status.

Weekly dialysis time was twelve hours in 83% of patients. The ultrafiltration rate was between 0.8 to 1.5 l per hour. Dialysis dose delivery was estimated from the urea Kt/V (urea clearance over time).

### 3.2. Data Collection

Demographic and clinical data such as age, gender, dialysis vintage, anthropometric parameters, cardiovascular risk factors, history of cardiovascular events, and use of nutritional supplementation were recorded. Body mass index (BMI) in kg/m^2^ was calculated as dry weight divided by height squared. This dry weight is regularly assessed and calculated for each patient on the basis of clinical status and bioimpedance analysis performed at the end of dialysis.

Predialysis and postdialysis systolic blood pressure (SBP) and diastolic blood pressure (DBP) were recorded with automated monitors for every dialysis session. Average SBP and DBP over a 1-month period were calculated.

Dialysis vintage was defined as the duration of time between the first day of HD treatment and December 31, 2015.

### 3.3. Laboratory Measures

All laboratory values were measured by automated and standardized methods, before the start of dialysis (on the day of the midweek dialysis session). Laboratory data refer to single measures.

Samples were collected for serum albumin, creatinine, and highly sensitive C-reactive protein (hsCRP) measurements. Serum albumin and serum creatinine (SCr) concentrations were determined.

The normalized protein catabolic rate (nPCR) [17] was used to assess the dietary protein intake.

NT-proBNP was assessed using a Siemens (DPC) Immulite 2000 chemiluminescence immunoassay based on N-terminal polyclonal sheep antibody.

### 3.4. Echocardiography

Standard transthoracic echocardiographic examination was performed by a cardiologist, who was blinded to the clinical data of the study subjects. All echocardiographic measurements were done according to the guidelines of the American Society of Echocardiography [9]. Left ventricular ejection fraction (LVEF) was calculated using the Simpson biplane method from 2 chambers and 4 chambers' apical views. Left ventricular mass (LVM) was calculated using the Devereux formula [18]. Left ventricular mass index (LVMI) was calculated as LVM/body surface area. Left ventricular hypertrophy (LVH) was defined by a LVMI > 134 g/m^2^ in men or >110 g/m^2^ in women.

### 3.5. Definition of Clinical Factors and Events

*Nutritional Status*. One component in each of the 4 categories of the wasting syndrome [3] was retained: serum albumin ≤ 38 g/L, BMI ≤ 23 Kg/m^2^, SCr ≤ 818 *μ*mol/L [2], and nPCR ≤ 0.8 g/kg/day.

Slight malnutrition was defined when one criterion for PEW was present, moderate malnutrition when two criteria were present, and severe malnutrition (PEW) in presence of three or four criteria [19].

- *Inflammation *was defined as a serum concentration of hsCRP of >5 mg/L.
- *Preexisting *cardiovascular (CV)* complications* included coronary event occurring before December* 2015.*
- Weight loss was defined as −5% over 3 months [3].
- Interdialytic weight gain (IDWG) was calculated by subtracting the postdialysis weight of previous HD session from the predialysis weight of the index HD session. The average IDWG of six previous sessions was considered.

### 3.6. Statistical Methods

Data are presented as percentages for categorical variables and as means ± standard deviations (SD) and medians (interquartile ranges, IQR) for continuous variables.

The chi-squared test and ANCOVA with adjustment for age, gender, or Mann–Whitney test were used to test percentage and mean differences between groups according to the presence or absence of high NT-proBNP levels. NT-proBNP values were logarithmically transformed to approach a normal distribution. The Pearson correlation test, adjusted for age and gender, was used to study the relationships between log NT-proBNP and other continuous variables.

The individuals were classified into 5 categories according to the number of criteria for PEW (ISRNM definition) with individuals exhibiting 0, 1, 2, 3, and 4 criteria.

We also used multivariate logistic regressions in the overall study population to determine the parameters associated with high NT-proBNP concentrations. In model 1, age/10 years, gender, predialysis SBP, dialysis vintage > 3 years, IDWG, diabetes, hsCRP > 5 mg/L, and nutritional status were included as covariates. In model 2, LVEF was included, in addition to the aforementioned covariates. The adjusted odds ratios and 95% confidence intervals (OR 95% CI) were provided.

Statistical analyses were performed by using IBM-SPSS statistical software package version 21 (IBM, Armonk, NY, USA). Statistical significance was defined as *P* < 0.05.

## 4. Results

Overall, 207 stable patients, undergoing HD at the dialysis centre, were included in the current study. The population was 54% male. Mean ± SD age was 64 ± 13 years and the mean dialysis vintage 7.2 ± 0.4 years. The major comorbidities were hypertension (90%), diabetes (41.5%), obesity (26.5%), and past history of coronary artery disease (CAD) (9.7%). Antihypertensive medications were prescribed to 82% of HD patients. All the patients had diuresis lower than 500 mL/day (i.e., no residual renal function).

Thirty-five patients (16.9%) had PEW (at least three parameters). Echocardiography was available for 159 patients for whom median [IQR] LVEF was 68% [63%–70%]. Among them, 13.8% had a LVEF lower than 60% and 3 patients (1.9%) had a LVEF lower than 40%. Characteristics of the patients are presented in Table 1.

NT-proBNP ranged from 125 to 33144 pg/mL with mean and median of 5243 ± 6573 and 2405 [1121–6243] pg/mL, respectively.

Since there was no threshold-consensus for HD patients, for the purpose of the study, participants with NT-proBNP ≥ 6243 pg/mL (75th percentile) were categorized as having high NT-proBNP levels.

Patients with high NT-proBNP levels were more likely to have higher dialysis vintage, higher frequencies of weight loss, low BMI (≤23 Kg/m^2^), low serum albumin levels (≤38 g/L), low serum creatinine levels (≤818 *μ*mol/L), low nPCR (≤0.8 g/kg/d), lower mean hemoglobin rate, higher frequencies of hsCRP > 5 (mg/L), nutritional supplementation, moderate malnutrition, and PEW. They also had lower frequency of diabetes, lower mean IDWG, and lower mean LVEF (Table 1). No significant difference was noted for age and frequencies of CAD history and of left ventricular hypertrophy.

Patients with PEW had a higher median NT-proBNP values and lower mean IDWG than those without PEW 6243 [1833–18721] versus 2132 [1100–5200] pg/mL, *P* = 0.002, and 1.7 ± 0.9 versus 2.5 ± 1.0 Kg, *P* < 0.001, respectively.

Median NT-proBNP in patients with and without diabetes was 2362 [1090–5245] versus 2453 [1162–7816], respectively, *P* = 0.219, and frequencies of PEW were 13% in patients with diabetes and 20% in those without diabetes, *P* = 0.183.

In 77 diabetic subjects with available glycated hemoglobin (A1CHb), there was no significant difference in mean A1CHb levels between those with (*n* = 11) and without (*n* = 66) high NT-proBNP levels: 7.04 ± 1.37% versus 7.03 ± 1.75%, respectively, *P* = 0.771.

### 4.1. Correlations of Log NT-proBNP with Clinical and Biological Parameters (Table 2)

There were positive correlations between log NT-proBNP and dialysis vintage (*r* = 0.18; *P* = 0.008), predialysis SBP (*r* = 0.18; *P* = 0.010), predialysis DBP (*r* = 0.20; *P* = 0.007), postdialysis SBP (*r* = 0.18; *P* = 0.009), and hsCRP (*r* = 0.21; *P* = 0.002) and negative correlations with BMI (*r* = −0.19; *P* = 0.005), nPCR (*r* = −0.15; *P* = 0.028), and LVEF (*r* = −0.24; *P* = 0.002).

### 4.2. Logistic Regression for High Values of NT-proBNP (≥6243 pg/mL)

In model 1 concerning 207 subjects, the following factors were identified: gender OR 2.80 (1.22–6.57), *P* = 0.010; dialysis vintage OR 3.80 (1.35–10.8), *P* = 0.012; hsCRP > 5 mg/L OR 3.90 (1.77–8.57), *P* = 0.001; and PEW OR 14.2 (3.25–62.4), *P* < 0.001. Having PEW (presence of 3 or 4 criteria) was associated with a 14-fold increase in the odds of having high NT-proBNP levels, Table 3.

In model 2 concerning 159 subjects with available echocardiographic data, independent factors for high values of NT-proBNP included gender OR 3.17 (1.18–8.49), *P* = 0.022; hsCRP > 5 mg/L OR 3.81 (1.45–10.0), *P* = 0.007; PEW OR 11.7 (2.01–64.2), *P* = 0.006; and LVEF OR 0.93 (0.88–0.98), *P* = 0.011.

The odds ratios for having moderate malnutrition (defined as the presence of 1 to 2 criteria) were nearly significant 3.28 (0.98–10.9), *P* = 0.052 in model 1, and 3.73 (1.83–16.7), *P* = 0.080 in model 2.

Of note, age, SBP, IDWG, and diabetes history were not independently associated with high levels of NT-proBNP.

### 4.3. Distribution of NT-proBNP and IDWG according to the Number of PEW Criteria

The five groups of subjects according to the number of criteria (0, 1, 2, 3, and 4) for PEW according to the ISRNM definition included 41 (19.8%), 67 (32.4%), 64 (30.9%), 21 (10.1%), and 14 (6.8%) subjects, respectively.

**Figure 1:**
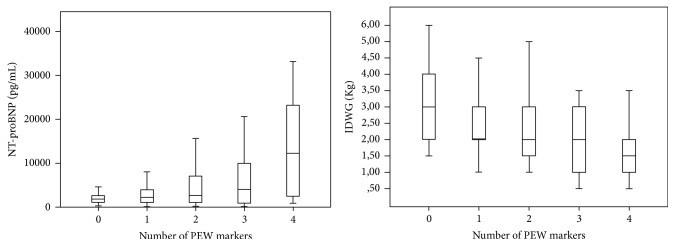
Distribution of NT-proBNP levels (*P* < 0.001) and IDWG (*P* < 0.001) according to the number of malnutrition criteria.

NT-proBNP (median [IQR]) concentrations were significantly higher when the number of malnutrition criteria was higher, for 0 criteria: 1858 [1143–2706] pg/mL, 1: 2276 [1092–4070] pg/mL, 2: 2676 [1045–7149] pg/mL, 3: 4025 [701–14340] pg/mL, and 4: 12289 [2507–23451] pg/mL (*P* < 0.001 for comparison of mean log NT-proBNP) (Figure 1).

Similar trends were found in both genders for median NT-proBNP and as follows: in men: for 0 criteria: 1883 pg/mL, 1: 1905 pg/mL, 2: 3001 pg/mL, 3: 12482 pg/mL, and 4: 14495 pg/mL and in women: for 0 criteria: 1505 pg/mL, 1: 2723 pg/mL, 2: 2662 pg/mL, 3: 3332 pg/mL, and 4: 6939 pg/mL.

The IDWG (median [IQR]) values decreased significantly with the number of malnutrition criteria: 0: 3 [2–4] Kg, 1: 2.1 [2-3] Kg, 2: 2 [1.5–3] Kg, 3: 2 [1–3] Kg, and 4: 1.25 [1-2] Kg (*P* < 0.001) (Figure 1).

## 5. Discussion

In the current study, in a cohort of Afro-Caribbean stable adult hemodialysis patients, we evaluated the association of NT-proBNP plasma levels and nutritional status using the ISRNM definition for protein energy wasting [3]. Our HD patients exhibited high levels of NT-proBNP as previously reported in ESRD patients [16, 20, 21]. NT-proBNP was associated with PEW and with left ventricular ejection fraction, independently of age, SBP, diabetes, hsCRP, and IDWG. In addition, we have also shown that NT-proBNP was higher and IDWG lower when the number of malnutrition criteria was higher. Our findings highlight the relationship between malnutrition and NT-proBNP concentrations.

### 5.1. NT-proBNP and Cardiometabolic Risk Factors

In the present study, we considered NT-proBNP values ≥ 6243 pg/mL (75th percentile) as the highest NT-proBNP levels, since there was no threshold-consensus for HD patients. In a recent study in 238 Japanese HD patients, NT-proBNP values ≥ 5760 pg/mL (higher tertile) were considered as the higher values [22]. Predialysis median NT-proBNP levels were previously found markedly elevated in HD patients, 4079 pg/ml [1893–15076] [14], compared to median population-based normal values, 20 pg/ml [10–30] [23].

The role of natriuretic peptides in cardiovascular homeostasis is well established. Brain natriuretic peptide is secreted by the heart mainly in response to the stretching of the myocardium induced by volume overload or in response to hypertrophy [24].

In this study, predialysis SBP was not associated with high levels of NT-proBNP in the multivariate logistic regression although high blood pressure is a common cause of increased left ventricular wall stress [25, 26]. The high frequencies of hypertension (90%) in this study population might contribute to these results.

The prevalence of abnormal left ventricular function (LVEF < 60%) was not high in this population (13.8%) and only 1.9% had a LVEF lower than 40%. In addition, no significant difference in frequencies of left ventricular hypertrophy was noted between NT-proBNP groups suggesting that factors other than cardiac status impact on NT-proBNP concentrations.

Insulin resistance has been associated with lower natriuretic peptide levels [27]. In this line, frequency of diabetes was higher in our patients with the lower levels of NT-proBNP (<6243 pg/mL) than in the others (46.5% versus 26.9%; *P* = 0.013). Recently, in patients without chronic kidney disease, prospective studies have shown that low levels of NT-proBNP are a positive predictor of incident type 2 diabetes [28, 29].

Inflammation (hsCRP > 5 mg/L) was associated with high NT-proBNP levels. Some authors described hsCRP as the most powerful cardiac biomarker for predicting all-cause of death when compared with NT-proBNP [16]. Inflammation also induces anorexia, reduces the effective use of dietary protein and energy intake, and augments protein catabolism [30].

### 5.2. NT-proBNP and PEW Components

In a previous study, malnutrition was accompanied by volume overload and was associated with increased NT-proBNP levels, independently of volume status [7].

Our patients with the highest levels of NT-proBNP exhibited higher frequencies of the four parameters used for the identification of PEW (ISRNM definition). Since there is no recognized threshold for low creatinine levels, we kept the creatinine value of our previous study in which patients who had SCr below 818 *μ*mol/L had a hazard ratio of death two times higher than those with SCr above this threshold [2]. The multivariate logistic regression showed that patients with PEW had a 14-fold higher odds of having high values of NT-proBNP compared with those with no criteria, independently of predialysis SBP, dialysis vintage, IDWG, and LVEF.

#### 5.2.1. Role of Body Mass Index

Several arguments are in favor of an important role of BMI and especially adipose tissue in the relationship between malnutrition and NT-proBNP levels. Negative linear relationships between BMI and plasma natriuretic peptide levels have been reported [31, 32]. In patients without renal insufficiency and without history of cardiomyopathy, obese patients have reduced concentrations of BNP and NT-proBNP compared to nonobese patients despite having elevated left ventricular end diastolic pressures [33].

In our study, patients with high levels of NT-proBNP were more likely to have a BMI ≤ 23 Kg/m^2^ (55.8%) than the others (29%) (Table 1) and also more likely to have had a weight loss that is also a malnutrition criterion according to the ISRMN definition [3]. In obese subjects undergoing weight loss surgery, weight loss was found associated with early increases in NT-proBNP concentrations [34].

Each of the three other malnutrition criteria used in our study (low nPCR values, low albumin, and low creatinine levels) was also associated with high NT-proBNP concentrations.

That could be explained by their own relationships with weight status. In fact, HD patients with greater protein and energy intakes usually have a greater BMI [35] and inversely. Hypoalbuminemia is the result of the combined effects of inflammation and inadequate protein and caloric intake [30] that may lead to low BMI. Creatinine levels are a surrogate of muscle mass in HD patients [36]. Anorexia and low nutrient intake may also lead to lower muscle mass, lower creatinine levels, and possibly lower BMI.

Weight gain and obesity have been associated with increased expression of natriuretic peptide receptors-C, in adipose tissue and increased degradation of natriuretic peptides [37]. NT-proBNP is essentially eliminated by the kidney, thus, in patient with low BMI or reduced adipose tissue, an increased synthesis or secretion of NT-proBNP by myocardial cells has been suggested [38]. Moreover, the existence of a heart-gut-brain axis, involving the ghrelin-appetite-hormone, was also evoked [39]. In this line, intravenous administration of BNP [34] or of human synthetic ghrelin [35] argued for an association between BNP concentrations and appetite regulation. The natriuretic peptides would participate in body weight regulation and energy homeostasis [35]. Thus NT-proBNP concentrations might reflect pathophysiological implication of BNP in these processes.

### 5.3. NT-proBNP, IDWG, and the Number of PEW Criteria

Interestingly, in our study, NT-proBNP concentrations were higher when the number of malnutrition criteria were higher while IDWG were lower (Figure 1). Interdialytic weight gain has been regarded as a surrogate of volume overload, in ESRD patients on HD but also as an index of good appetite and nutritional status [40, 41]. Our results concerning NT-proBNP concentrations suggest that the heart-gut-brain axis is particularly stimulated when the degree of malnutrition is high.

This study has some limitations including the single measurement of NT-proBNP and other laboratory parameters and also the lack of other anthropometric parameters (such as mid-arm circumference) and other markers of malnutrition. Results of bioelectrical impedance were not available and the hydration status was not taken into account. The cross-sectional design did not let us draw any causality link.

But our study also has several strengths. Data were obtained in a homogenous population of Afro-Caribbean subjects and it is known that measures of serum creatinine and other nutritional markers may vary according to ethnic groups. There was no difference in dialysis quality and age between NT-proBNP groups whereas NT-proBNP concentrations were reported to increase with age. There was also no bias in relation to type of dialysis or dialysis membrane since dialysis modalities were identical for all subjects and performed with synthetic high flux membranes.

## 6. Conclusion

The results of the present study confirm the association between malnutrition and NT-proBNP concentrations. In addition we demonstrated that NT-proBNP concentrations are higher when the number of malnutrition criteria is higher. Since high NT-proBNP levels and a worse nutritional status are both prognostic factors of survival, in dialysis patients, high NT-proBNP levels must draw attention to cardiac function but also to nutritional status.

## Figures and Tables

**Table 1:** Characteristics of hemodialysis patients according to NT-proBNP levels.

		All patients *N* = 207	NT-proBNP (pg/mL) *N* = 207		
			<6243 *n* = 155	≥6243 *n* = 52	*P*
Age (y)	207	64 ± 13	63 ± 14	65 ± 12	*0.382*
Dialysis vintage (y)	207	7.2 ± 0.4	6.9 ± 0.8	7.9 ± 0.9	*0.342*
Dialysis vintage ≥ 3 y (%)	207	73.4	69.0	86.5	***0.013***
Sex (men)	207	54.1	51.0	63.5	*0.118*
Diabetes (%)	207	41.5	46.5	26.9	***0.013***
Previous CAD (%)	207	9.7	9.0	11.5	*0.597*
Hypertension (%)	207	89.9	89.7	90.4	*0.884*
Predialysis SBP (mmHg)	207	146 ± 24	145 ± 24	149 ± 26	*0.280*
Predialysis DBP (mmHg)	207	81 ± 17	80 ± 14	83 ± 17	*0.091*
Postdialysis SBP (mmHg)	207	137 ± 26	135 ± 26	143 ± 27	***0.048***
Postdialysis DBP (mmHg)	207	76 ± 16	75 ± 16	77 ± 17	*0.453*
Hemoglobin (g/dL)	207	11.8 ± 1.5	11.9 ± 1.4	11.3 ± 1.6	***0.008***
Serum sodium (mmol/L)	207	139 ± 3	139 ± 2	138 ± 3	*0.184*
IDWG (Kg)	207	2.3 ± 1.0	2.4 ± 1.1	2.1 ± 0.8	***0.014***
KT/V	207	1.3 ± 0.2	1.3 ± 0.2	1.4 ± 0.2	*0.475*
Nutritional supplementation	207	26.6	22.6	38.5	***0.025***
*Nutritional parameters*					
Body mass index (Kg/m^2^)	207	26.1 ± 6.7	26.9 ± 6.9	23.8 ± 5.2	***0.004***
Body mass index ≤ 23 Kg/m^2^ (%)	207	35.7	29.0	55.8	***<0.001***
Serum albumin (g/L)	207	38.2 ± 4.5	38.5 ± 4.5	37.3 ± 4.4	*0.095*
Serum albumin ≤ 38 g/L (%)	207	47.3	43.2	59.6	***0.041***
Serum creatinine (*µ*mol/L)	207	884 ± 278	915 ± 275	793 ± 268	***0.001***
Serum creatinine ≤ 818 *µ*mol/L (%)	207	41.1	34.2	61.5	***0.001***
NPCR (g/kg/D)	207	0.94 ± 0.21	0.96 ± 0.21	0.87 ± 0.21	***0.010***
NPCR ≤ 0.8 g/kg/D (%)	207	27.5	23.9	38.5	***0.042***
Malnutrition (≥1 factor) (%)	207	80.2	76.1	92.3	***0.011***
PEW (≥3 factors) (%)	207	16.9	11.0	34.6	***<0.001***
hsCRP > 5 (mg/L)	207	48.5	40.6	71.2	***<0.001***

Echocardiographic parameters		All patients *N* = 159	NT-proBNP (pg/mL)		
			<6243 *N* = 122	≥6243 *N* = 37	*P*

LVEF (%)	159	65.1 ± 8.5	66.3 ± 7.3	60.9 ± 10.9	***<0.001***
LVEF < 60 (%)	159	13.8	10.7	24.3	***0.035***
Left ventricular hypertrophy (%)	159	40.3	38.5	45.9	*0.420*

Data in this table are presented as column percentages or mean ± SD. Significant *P* values are in bold.

**Table 2:** Correlations between log NT-proBNP and clinical, biological, and echocardiographic parameters.

	*n*	*r*	*P*
Age ( y)	207	0.03	*0.600*
Dialysis vintage (y)	207	0.18	***0.008***
Kt/V	207	0.09	*0.176*
IDWG	207	−0.11	*0.121*
Predialysis SBP (mmHg)	207	0.18	***0.010***
Predialysis DBP (mmHg)	207	0.20	***0.007***
Postdialysis SBP (mmHg)	207	0.18	***0.009***
Postdialysis DBP (mmHg)	207	0.11	***0.143***
hsCRP (mg/L)	207	0.21	***0.002***
Hemoglobin (g/dL)	207	−0.15	***0.032***

*Nutritional parameters*			
Body mass index (Kg/m^2^)	207	−0.19	***0.005***
Serum albumin (g/L)	207	−0.09	*0.197*
Serum creatinine (*µ*mol/L)	207	−0.14	*0.078*
NPCR (g/kg/D)	207	−0.15	***0.028***

*Echocardiography*			
LVEF (%)	159	−0.24	***0.002***

Correlations adjusted for age and sex.

**Table 3:** Multivariate logistic regression for high values of NT-proBNP (≥6243 pg/mL).

	Model 1 *N* = 207		Model 2 *N* = 159	
	OR (95% CI)	*P*	OR (95% CI)	*P*
Age/10 y	1.01 (0.99–1.03)	*0.831*	1.01 (0.98–1.04)	*0.420*
Sex (M)	2.80 (1.22–6.57)	***0.015***	3.17 (1.18–8.49)	***0.022***
Predialysis SBP	1.01 (0.99–1.02)	*0.175*	1.01 (0.99–1.02)	*0.310*
Dialysis vintage > 3 y (yes/no)	3.80 (1.35–10.8)	***0.012***	2.40 (0.80–7.21)	*0.118*
IDWG (Kg)	0.68 (0.43–1.08)	*0.107*	0.57 (0.32–1.01)	*0.050*
Diabetes (Yes/No)	0.50 (0.22–1.13)	*0.099*	0.46 (0.18–1.17)	*0.110*
hsCRP > 5 mg/L (Yes/No)	3.90 (1.77–8.57)	***0.001***	3.81 (1.45–10.0)	***0.007***
Moderate malnutrition/normal nutritional status	3.28 (0.98–10.9)	*0.052*	3.73 (1.83–16.7)	*0.081*
Severe malnutrition (PEW)/normal nutritional status	14.2 (3.25–62.4)	***<0.001***	11.7 (2.01–64.2)	***0.006***
Left ventricular ejection fraction %	—	—	0.93 (0.88–0.98)	***0.011***
